# Beta-Lactam Sensitive Bacteria Can Acquire ESBL-Resistance via Conjugation after Long-Term Exposure to Lethal Antibiotic Concentration

**DOI:** 10.3390/antibiotics9060296

**Published:** 2020-06-02

**Authors:** Pilvi Ruotsalainen, Cindy Given, Reetta Penttinen, Matti Jalasvuori

**Affiliations:** 1 Nanoscience Center, Department of Biological and Environmental Science, University of Jyväskylä, P.O. Box 35, FI-40014 Jyväskylä, Finland; pilvi.ruotsalainen@gmail.com (P.R.); cindy.j.given@jyu.fi (C.G.); reetta.k.penttinen@jyu.fi (R.P.); 2 Faculty of Science and Engineering, Department of Biology, University of Turku, FI-20014 Turku, Finland

**Keywords:** antibiotic resistance, Extended-spectrum beta-lactamase, evolutionary rescue, conjugative plasmid

## Abstract

Beta-lactams are commonly used antibiotics that prevent cell-wall biosynthesis. Beta-lactam sensitive bacteria can acquire conjugative resistance elements and hence become resistant even after being exposed to lethal (above minimum inhibitory) antibiotic concentrations. Here we show that neither the length of antibiotic exposure (1 to 16 h) nor the beta-lactam type (penam or cephem) have a major impact on the rescue of sensitive bacteria. We demonstrate that an evolutionary rescue can occur between different clinically relevant bacterial species (*Klebsiella pneumoniae* and *Escherichia coli*) by plasmids that are commonly associated with extended-spectrum beta-lactamase (ESBL) positive hospital isolates. As such, it is possible that this resistance dynamic may play a role in failing antibiotic therapies in those cases where resistant bacteria may readily migrate into the proximity of sensitive pathogens. Furthermore, we engineered a Clustered Regularly Interspaced Short Palindromic Repeat (CRISPR)-plasmid to encode a guiding CRISPR-RNA against the migrating ESBL-plasmid. By introducing this plasmid into the sensitive bacterium, the frequency of the evolutionarily rescued bacteria decreased by several orders of magnitude. As such, engineering pathogens during antibiotic treatment may provide ways to prevent ESBL-plasmid dispersal and hence resistance evolution.

## 1. Introduction

Resistance to antibiotics forms a notable burden to health care. While the evolution of resistance may appear as a seemingly simple evolutionary process, i.e., exposure to antibiotics selects for resistant mutants [1], the actual emergence of new resistant pathogens and the maintenance of resistance may be a result of relatively complex interbacterial interactions [2]. This is especially relevant for horizontally transferred resistance genes that reside in conjugative plasmids [3].

β-lactams are antibiotics that inhibit the synthesis of the bacterial cell wall. Given their minimal side effects in humans, they are one of the most widely applied antibiotics in clinical care [4]. Respectively, extended-spectrum β-lactamases (ESBLs) are enzymes that can hydrolyze and hence inactivate a wide range of different β-lactams [5]. Notably, the presence of ESBL-producing bacteria allows sensitive “cheaters” to survive in a shared environment even in lethal antibiotic concentrations [6,7]. Resistant bacteria may be a part of the commensal flora and pose no threat in itself, therefore the resistance profile of the pathogen may not necessarily determine whether or not the applied antibiotic is effective [7,8,9]. This is troublesome since ESBL-carriage, i.e., the asymptomatic colonization of the gut by ESBL bacteria, has been continuously increasing both in hospital settings and in the community [10]. As such, a more complete understanding of the evolutionary dynamics of resistances during antibiotic treatment would help us to more accurately evaluate the potential risks that may be incurred from the carriage of resistant strains.

Sensitive bacteria may acquire resistance via horizontal gene transfer (HGT) even after being exposed to lethal beta-lactam concentrations [7,11]. The physiological factors affecting the efficacy of β-lactams are currently being studied by numerous groups and can include cell-wall-lacking spheroplasts and dormant persisters [12,13]. Nevertheless, for ESBL-carriers, the evolutionary rescue may form an additional threat as the treatment of initially sensitive Gram-negative pathogens can be nullified by an accidental transfer of an ESBL-plasmid harboring bacterium from the gut flora to the site of infection.

There is variability in the potential for different conjugative plasmids with various Inc. (incompatibility)-groups and ESBL-genes to rescue susceptible bacteria [11]. It is also dependent on the antibiotic concentration and resource availability. Rescue may occur even in antibiotic levels that exceed the minimum inhibitory concentrations by an order of magnitude, therefore the increase in dosage does not seem to provide a straightforward solution to prevent the transfer [14]. Furthermore, sensitive bacteria will survive and become resistant when the resistant bacteria are subsequently introduced into their environment [7,11]). However, several factors potentially affecting the rescue have not been studied yet. Namely, it is unclear what the time-window is in which the transfer of an ESBL-plasmid from a harmless bacterium may still restore the growth of the sensitive pathogen, what the effect of different types of β-lactams is, whether the prevailing temperature (and hence bacterial metabolic rate) plays a part, and/or whether the species of the sensitive pathogen is relevant to the rescue. Here, we investigate how these factors affect the evolutionary rescue via a conjugative ESBL-plasmid of clinical origin. Understanding the role of these factors assists in determining how and when the ESBL-carriage status needs to be taken into account.

## 2. Results and Discussion

**Figure 1:**
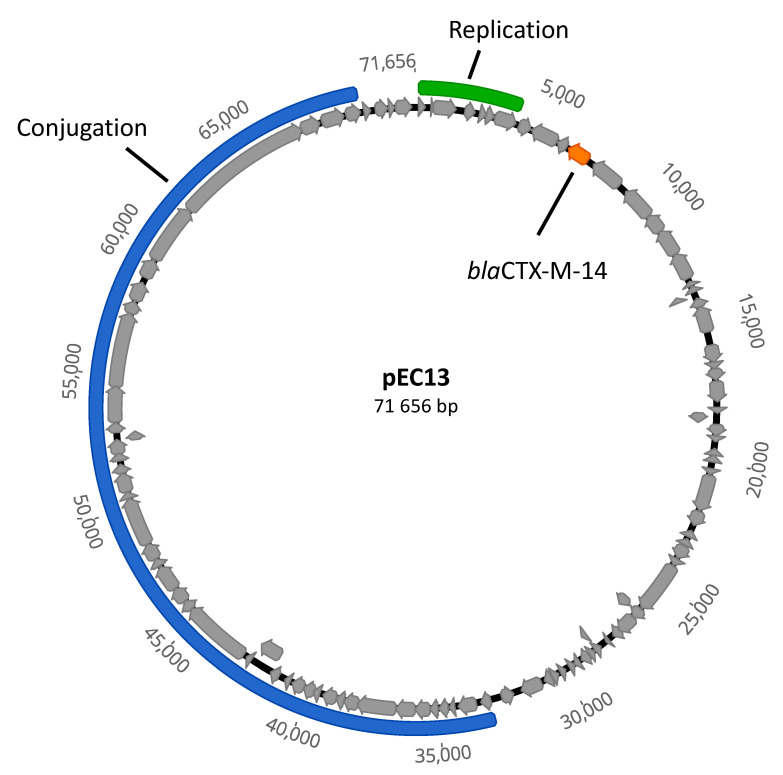
Plasmid map of conjugative pEC13. The predicted coding regions are marked in grey. Regions involved in the conjugation (blue), replication (green) and antibiotic resistance (orange) are marked.

In this study, we selected a previously characterized plasmid pEC13 to investigate the evolutionary rescue in lethal β-lactam concentrations. pEC13 is a 71 kb conjugative IncFII-type plasmid, which originates from a patient-derived ESBL *Escherichia coli* (Figure 1; [11]) and carries a commonly circulating ESBL-resistance gene, *blaCTX-M-14* [10]. The majority of the pEC13-like plasmids in databases have been isolated from *E. coli.* Yet, there are multiple closely related plasmid sequences also in *Klebsiella pneumoniae*, *Salmonella enterica*, *Citrobacter* sp., *Shigella sonnei* and *Shigella flexneri* hosts, indicating that these plasmids may serve as potential agents mediating the interspecific resistance exchange.

**Figure 2:**
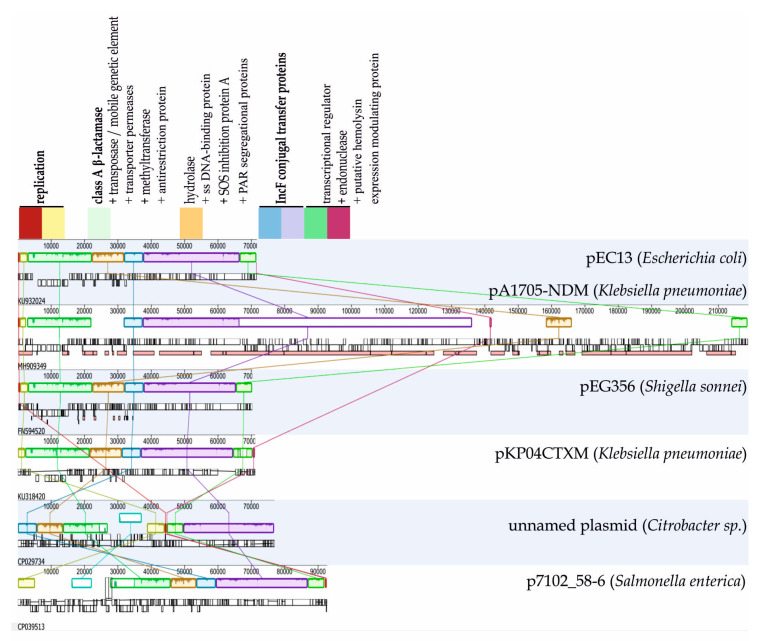
Comparison of pEC13 to related plasmids in other Enterobacteriaceae. The genetic regions are color-coded according to their predicted function (the exact gene contents within the regions vary between plasmids). Plasmids with relatively similar genetic contents and synteny to pEC13 are present in various bacterial species, hence the interspecific evolutionary rescue via horizontal gene transfer.

While pEC13 carries a single resistance gene and hence only provides resistance to non-carbapenem β-lactams, the highest similarity to pEC13 (for the matching regions) was with a *K. pneumoniae* plasmid pA1705-NDM (99.85%, GenBank id MH909349) encoding NDM-1, OXA-1 and CTX-M-14 β-lactamases (where NDM-1 is also able to hydrolyse carbapenems) along with resistances to fluoroquinolone, aminoglycoside, phenicol, rifampicin, sulphonamide, and tetracycline. As such, related plasmids can also confer resistances to multiple types of antibiotics. Plasmids such as pA1705-NDM may be of notable clinical relevance given that evolutionary rescue may also nullify any subsequent treatment attempts with alternative antibiotics once the initial failure occurs with β-lactams. PA1705-NDM plasmid, however, is over three times the length of pEC13. This genomic expansion appears to be due to the accumulation of several mobile elements and resistance cassettes in the plasmid backbone, and hence a direct comparison with the resistance dynamics observed for pEC13 in this study should be made with caution. Still, other similar plasmids in other species appear to have retained their size as well as gene and operon synteny with pEC13 (Figure 2), and therefore pEC13 appears to provide a relevant proxy for ESBL-plasmids that may disperse between different species of *Enterobacteriaceae* during the exposure to β-lactams.

**Figure 3:**
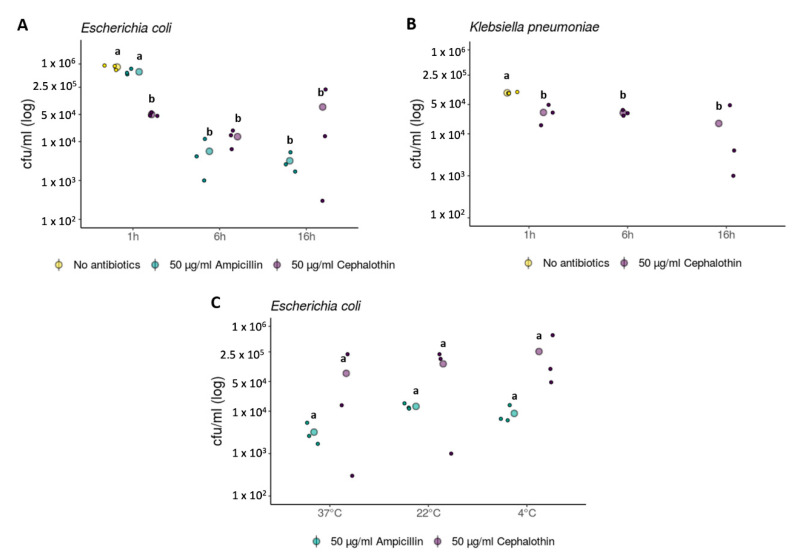
Evolutionary rescue of *Escherichia coli* and *Klebsiella pneumoniae* after differing exposure times and at different temperatures. The big dots represent the evolutionary rescue presented as the mean cell density in colony forming units (cfu)/mL of formed transconjugants after the exposure to either ampicillin (50 µg/mL) or cephalothin (50 µg/mL) and the introduction of a rescuing strain harboring beta-lactamase resistance gene against these antibiotics in conjugative plasmid pEC13. The small dots represent individual data points per treatment. For reference, the number of transconjugants in the absence of antibiotics was measured at the 1 h time point. Statistical analysis and standard deviations are presented in Table S1. Letters indicate the results from Tukey’s “Honest Significant Difference” test. Groups indicated by the same letter do not differ significantly. Different exposure times to antibiotics were tested for (**A**) Escherichia coli and (**B**) Klebsiella pneumoniae. (**C**) The effect of temperature (37, 22 or 4 °C) on the evolutionary rescue of *E. coli* was measured after 16 h exposure to ampicillin and cephalothin.

We examined the transfer of pEC13 between two *E. coli* strains, as well as between *E. coli* and *K. pneumoniae*. Ampicillin or cephalothin sensitive *E. coli* and cephalothin sensitive *K. pneumoniae* strains were exposed to lethal antibiotic concentrations (50 μg/mL) for 1, 6, and 16 h before resistant *E. coli* harboring pEC13 plasmid was introduced into the environment (Figure 3A,B). New resistant bacteria emerged in all treatments (see Table S1). The number of evolutionarily rescued *E. coli* decreased with ampicillin and cephalothin in relation to longer exposure periods (Figure 3A; Table S2); a statistical difference was observed between 1 h exposure to antibiotics compared to 6 h and between 1 and 16 h (Tukey HSD, *p* < 0.001), but not between 6 and 16 h (Tukey HSD, *p* = 0.76). In *K. pneumoniae*, the exposure time had no effect on the evolutionary rescue (*p* = 0.47; Figure 3B; Table S3). The antibiotic type used had no significant effect on the evolutionary rescue of *E. coli* (*p* = 0.055). However, given the notable variance in some measurements, the statistical significance needs to be taken cautiously. Nevertheless, both strains were able to acquire pEC13 even after 16 h and become completely resistant to these antibiotics. The possibility that the result may be due to spontaneous rifampicin-resistant pEC13 donors was ruled out by testing the chloramphenicol resistance of the rescued bacteria, as only the pEC13 donor carried a non-mobilizable chloramphenicol resistance conferring plasmid pSU19. Furthermore, we observed that the viable *E. coli* could be isolated after one hour antibiotic exposure even when no donor was introduced. This suggests that cell-wall-lacking spheroplasts or otherwise antibiotic-withstanding phenotypes [15,16]. may persist in the presence of the tested antibiotics and still acquire the conjugative plasmid as the opportunity occurs. The media used (LB -broth) is slightly hypotonic (85.5 mM) compared to isotonic media with the molarity of physiological saline (154 mM), probably allowing the bacteria to retain osmotic stability. Bacteria are generally able to tolerate slight changes in the osmolarity of the environment even in the absence of a supporting cell-wall by adjusting their metabolism through pressure-sensitive ion channels [17] and by regulating their fatty acid synthesis to maintain lipid membranes [15].

The prevailing temperature has a substantial effect on the rate at which bacteria replicate. Given that replication itself is dependent on the generation of new cell walls, it is possible that lower temperatures hinder the efficacy of cell-wall-targeting antibiotics and thus allow more bacteria to acquire the plasmid. As such, we tested whether temperature has an effect to bacterial survival via evolutionary rescue in the presence of antibiotics. *E. coli* was exposed to cephalothin and ampicillin at 4 °C, room temperature (22 °C), and 37 °C for 16 h before the introduction of the pEC13-harboring strain (Figure 3C; Table S4). We found no significant difference in the rescued cells between cephalothin and ampicillin (*p* = 0.055; Table S5), nor the temperatures used (*p* = 0.56; Table S5). Nevertheless, this suggests that the persisting cells are already present in the population before the exposure to antibiotics. As such, the migration of bacteria harboring conjugative resistance plasmids can also rescue susceptible bacteria in the environmental reservoirs and hospital areas that contain notable beta-lactam pollution but where bacterial growth may be reduced. Qualitatively, the results show that a rescue can occur even after 16 h exposure to commonly used antibiotics, between different bacterial species, and within a wide temperature regime.

We studied whether the evolutionary rescue of the sensitive bacteria may be prevented by introducing them to a CRISPR-Cas9 plasmid that targets the β-lactamase gene of pEC13. In other words, the incoming pEC13 should be degraded by Cas9 in the persisting clones that remain viable in the presence of the antibiotics. We first ensured that pEC13 is not able to mobilize the CRISPR plasmid by aligning the OriT and OriR sequences from pEC13 with pCas9-plasmid and establishing that they are absent in pCas9-gRNA. Indeed, the presence of a CRISPR-plasmid significantly reduced the number of transconjugants on the double-antibiotic (chloramphenicol and ampicillin) plates by at least four orders of magnitude after 2 h exposure to ampicillin (Table 1). This at least reveals the possibility that potential pathogens would be less likely to acquire resistances if they were, in one way or another, introduced to β-lactamase targeting CRISPR-elements before or simultaneously with an antibiotic treatment. Hypothetically, bacteriophages could serve as an imaginable way to deliver CRISPR-systems to bacteria, e.g., in infected wounds [18]. Alternatively, conjugative plasmids can be engineered to transfer CRISPR-systems [19] and hence utilized within probiotic strains to prophylactically limit the abundance of ESBL-plasmids in gut flora, and therefore the rescue events from taking place.

To conclude, evolutionary rescue by horizontal gene transfer is an event where susceptible bacteria may become resistant to antibiotics ‘on the fly’ after the beginning of the treatment. Here we demonstrate that the rescue of susceptible *E. coli* and *K. pneumoniae* strains may occur even after 16 h exposure to both ampicillin and cephalothin. The applicability of this observation to real life systems must be taken cautiously. Yet, it is possible that if any suspected pathogen is determined to be sensitive to β-lactams, the protection of the infected site from the migration of (even harmless) ESBL-bacteria may be crucial for the treatment outcome. Preventive measurements that block plasmid-conjugation and/or maintenance [2,3,14] or nick resistance genes (such as the aforementioned CRISPR-systems) are still in the very early stage of development for constraining evolutionary rescue and/or ESBL-carriage but may be worth considering in the future.

## 3. Materials and Methods

### 3.1. Bioinformatic Analyses

To explore the prevalence of pEC13-like plasmids, highly similar matches were searched with a NCBI BLAST Megablast-search using a nucleotide collection database (nr/nt) with a Max E-value of 10, a word size of 28, a and scoring (Match Mismatch) of 1–2. The search was performed with Geneious 11.1.5 (Biomatters Ltd.; Auckland, New Zealand). The conserved gene clusters, their arrangement, and orientation were studied for the plasmid matches from various *Enterobacteriaceae* species with a grade of 70% or above (a total of 40 plasmids of which six non-*E. coli* plasmids are presented in the study) using a whole genome alignment tool with a progressive Mauve algorithm with default settings [20]. To ensure that the pCas9-gRNA plasmid is not transmissible by pEC13, oriTFinder [21] was used to detect the origin of the transfer (OriT) region from pEC13. The origin of the replication (OriR) region of pEC13 was identified with Ori-Finder 2 [22].

### 3.2. Evolutionary Rescue Experiments

Conjugation assays were performed to study the impact of a delayed introduction of an ESBL-resistant donor to a culture of antibiotic sensitive bacteria under a lethal antibiotic concentration. As a conjugative plasmid, we used pEC13, IncFII plasmid encoding CTX-M-14 β-lactamase [11], GenBank id KU932024.1). *Escherichia coli* K-12 JM109(pEC13)(pSU19) was used as a donor for two different recipients: *Escherichia coli* K-12 HMS174 and *Klebsiella pneumoniae* DSM681*^AmpR,RifR^*. DSM681 carries a gene for SHV-1 beta-lactamase, which makes the strain resistant to ampicillin, but not cephalothin. The DSM681 (or ATCC 10031) genome sequence is available at genomes.atcc.org for registered users. No other beta-lactamase genes are present in the DSM681 genome as determined with ResFinder-3.2 [23]. We also tested the conjugation of pEC13 from *E. coli* to a rifampicin resistant mutant of *Salmonella enterica* serovar Typhimurium SL5676, but no transconjugants were observed and hence it was omitted from further experiments.

The donor and recipient strains were grown to a carrying capacity in overnight culture (37 °C, 220 rpm) in a Luria Bertani Lennox-broth (LB; Lennox 1955) [24] with an appropriate antibiotic selection: donor in 150 µg/mL ampicillin and recipients in 50 µg/mL rifampicin. The cell density of overnight cultures was determined by serial dilutions plated on 1% LB agar plates and incubated overnight at 37 °C. Approximately 5.5 × 10^6^ and 7.25 × 10^6^ colony forming units (CFU) of sensitive recipient bacteria, HMS174 and DSM681, respectively, were first introduced to 5 mL of LB-broth supplemented with either 50 µg/mL of ampicillin or cephalothin. In a previous study, this concentration was shown to be clearly above MIC, as 15 µg/mL was already lethal to the same strains used here [11]. DSM681 was exposed only to cephalothin due to its chromosomal resistance to ampicillin. After different exposure times (1, 6, and 16 h) at 37 °C, the donor bacteria (4.1 × 10^6^ CFU) were added to the culture. These time points provide an estimate as to whether the protection of the sensitive bacterium from the migration of resistant bacteria early on after antibiotic administration can have an effect. As a control treatment, this assay was also done without antibiotic selection for the 1-h time point. This ‘no-antibiotics’ assay represents just the plasmid conjugation and hence not the evolutionary rescue. For the HMS174 recipient, two additional temperatures (4 and 22 °C) for the 16 h timepoint were examined. Each assay was performed at a minimum in triplicates. After the introduction of the resistant ESBL-donor, the co-culture was incubated overnight (37 °C, 220 rpm), after which the number of transconjugants was determined by plating on 1% LB-agar plates with the appropriate antibiotics: rifampicin (50 µg/mL) and either ampicillin 150 µg/mL (for HMS174) or cephalothin 50 µg/mL (for DSM681). Before the introduction of the donor, a 500 µL sample was taken from the 16 h exposure experiments to determine the presence of surviving bacteria. The sample was pelleted by centrifugation (8000 rpm, 8 min), the supernatant was removed carefully, and the pellet was resuspended in 100 µL of sterile water before being plated on the LB-agar without antibiotics. The presence of the surviving bacteria was quantified by observing the colonies on the plates after overnight cultivation at 37 °C.

We also evaluated the efficacy of CRISPR-Cas9-encoding plasmid to prevent an evolutionary rescue via horizontal gene transfer. The CRISPR-plasmid used (pCas9-gRNA) targets a conserved region in the *bla*CTX-M-14 gene with the spacer sequence CCGCTGGTTCTGGTGACCTATTT (described in detail in Ruotsalainen et al., 2019). The original pCas9 plasmid was a gift from Luciano Marraffini (Addgene plasmid #42876) and confers resistance to chloramphenicol. As a control, we used the same plasmid without the targeting spacer (pCas9-CTRL). These plasmids were transformed into *E. coli* DH5α strains by electroporation. HMS174 (pEC13) was used as a donor for the rescue experiments. A total of 5 µL of overnight grown DH5α (pCas9-gRNA) and DH5α (pCas9-CTRL) were exposed to 50 µg/mL ampicillin in 5 mL of LB-medium in 37 °C for 2 h before adding 5 µL of HMS174(pEC13). This combination was cultivated for 16 h (37 °C, 220 rpm) and plated on LB-agar with ampicillin (450 µg/mL) and chloramphenicol (75 µg/mL). Increased antibiotic concentrations were used in order to inhibit the appearance of false positive rescued colonies emerging in the plating of higher bacterial densities.

### 3.3. Statistical Analyses

To determine the effect of the selected antibiotics and the temperature on the evolutionary rescue of antibiotic sensitive bacteria, a two-way between-groups analysis of variance (ANOVA) with Tukey HSD as post-hoc comparisons was conducted using RStudio Cloud service. Three treatments were regarded in the analysis: the type of β-lactams antibiotics, time points, and temperatures.

## 4. Conclusions

Dispersal of antibiotic resistance genes plays a notable part in the emerging resistance crisis. ESBL-genes often reside in conjugative plasmids and may spread interspecifically between bacteria. Here, we show that antibiotic susceptible *E. coli* and *K. pneumoniae* exposed to inhibitory concentrations of beta-lactam antibiotics cephalotin and ampicillin for several hours can still become resistant by acquiring a conjugative plasmid from migrating resistant bacteria. 

## Figures and Tables

**Table 1:** The evolutionary rescue of CRISPR-plasmid harboring antibiotic susceptible *E. coli*.

Beta-Lactam Susceptible Strain	Replicate	Survivors (cfu/mL)
	1	<200
*E. coli* DH5α (pCas9-gRNA)	2	<200
	3	<200
	1	1.52 × 10^6^
*E. coli* DH5α (pCas9-CTRL)	2	1.76 × 10^6^
	3	2.12 × 10^6^

## Supplementary Materials

The following are available online at https://www.mdpi.com/2079-6382/9/6/296/s1, Table S1: Descriptive statistics on the evolutionary rescue of *E. coli* and *K. pneumoniae* under different β-lactams (50 µg/mL ampicillin and 50 µg/mL cephalothin) and exposure time (1 h, 6 h, and 16 h). Table S2: Analysis of Variance, test effect of β-lactams (50 µg/mL ampicillin and 50 µg/mL cephalothin) and exposure time (1 h, 6 h, and 16 h) on the evolutionary rescue of *E. coli*., Table S3, Analysis of Variance, test effect of β-lactams (50 µg/mL ampicillin and 50 µg/mL cephalothin) and exposure time (1 h, 6 h, and 16 h) on the evolutionary rescue of *K. pneumoniae*. Table S4: Descriptive statistics on the evolutionary rescue of *E. coli* under different β-lactams (50 µg/mL ampicillin and 50 µg/mL cephalothin) and temperature (37 °C, 22 °C, and 4 °C), Table S5: Analysis of Variance, test effect of β-lactams (50 µg/mL ampicillin and 50 µg/mL cephalothin) and temperature (37 °C, 22 °C, and 4 °C) on the evolutionary rescue of *E. coli*.
