# Supplementary material for: Beta-Lactam Sensitive Bacteria Can Acquire ESBL-Resistance via Conjugation after Long-Term Exposure to Lethal Antibiotic Concentration
Source: Antibiotics (Basel). 2020 Jun 2;9(6):296. doi: 10.3390/antibiotics9060296 (PMC7345503; doi:10.3390/antibiotics9060296)
Supplement: Supplementary file 1 [file antibiotics-09-00296-s001.pdf]

# Beta-lactam sensitive bacteria can acquire ESBL-resistance via conjugation after long-term exposure to lethal antibiotic concentration

Pilvi Ruotsalainen[1], Cindy Given[1], Reetta Penttinen[1,2] and Matti Jalasvuori[1]*

[1]University of Jyväskylä, Department of Biological and Environmental Science, Nanoscience Center, Finland
[2]University of Turku, Department of Biology, Faculty of Science and Engineering, Finland
* Correspondence: Matti Jalasvuori, Ambiotica Building, Survontie 9C, Department of Biological and Environmental Science, P.O.Box 35, FIN-40014, University of Jyväskylä, Finland E-mail: matti.jalasvuori@jyu.fi Phone: +358 50 4135092

## SUPPLEMENTARY DATA

### Supplementary table 1

Descriptive statistics on the evolutionary rescue of *E. coli* and *K. pneumoniae* under different β-lactams (50 µg/mLL ampicillin and 50 µg/mL cephalothin) and exposure time (1h, 6h, and 16h).

| Bacteria | β-lactams | Exposure time | Mean | SD | N |
|---|---|---|---|---|---|
| *E. coli* | No antibiotics | 1h | 8.30E+05 | 1.14E+05 | 3 |
| | 50 µg/mL ampicillin | 1h | 6.27E+05 | 1.10E+05 | 3 |
| | | 6h | 5.65E+03 | 5.53E+03 | 3 |
| | | 16h | 3.21E+03 | 1.89E+03 | 3 |
| | 50 µg/mL cephalothin | 1h | 5.00E+04 | 6.08E+03 | 3 |
| | | 6h | 1.34E+04 | 6.53E+03 | 3 |
| | | 16h | 7.80E+04 | 1.23E+05 | 3 |
| *K. pneumoniae* | No antibiotics | 1h | 9.47E+04 | 3.79E+03 | 3 |
| | 50 µg/mL cephalothin | 1h | 3.23E+04 | 1.65E+04 | 3 |
| | | 6h | 3.17E+04 | 5.03E+03 | 3 |
| | | 16h | 1.77E+04 | 2.63E+04 | 3 |

### Supplementary table 2

Analysis of Variance, test effect of β-lactams (50 µg/mL ampicillin and 50 µg/mL cephalothin) and exposure time (1h, 6h, and 16h) on the evolutionary rescue of *E. coli*.

| | df | Sum Sq | Mean Sq | F value | Pr(>F) |
|---|---|---|---|---|---|
| antibiotics | 2 | 1.3839e+12 | 6.9193e+11 | 120.459 | 1.507e-09 *** |
| time | 2 | 3.9540e+11 | 1.9770e+11 | 34.419 | 3.938e-06 *** |
| antibiotics x time | 2 | 3.8526e+11 | 1.9263e+11 | 33.535 | 4.580e-06 *** |
| Residuals | 14 | 8.0417e+10 | 5.7441e+09 | | |

*** p < 0.001

**Supplementary table 3**

Analysis of Variance, test effect of β-lactams (50 μg/mL ampicillin and 50 μg/mL cephalothin) and exposure time (1h, 6h, and 16h) on the evolutionary rescue of *K. pneumoniae*.

| | df | Sum Sq | Mean Sq | F value | Pr(>F) |
|---|---|---|---|---|---|
| antibiotics | 1 | 1.0235e+10 | 1.0235e+10 | 40.7621 | 0.0002126 *** |
| time | 2 | 4.1156e+08 | 2.0578e+08 | 0.8196 | 0.4744719 |
| Residuals | 8 | 2.0087e+09 | 2.5108e+08 | | |

*** $p < 0.001$

**Supplementary table 4**

Descriptive statistics on the evolutionary rescue of *E. coli* under different β-lactams (50 μg/mL ampicillin and 50 μg/mL cephalothin) and temperature (37°C, 22°C, and 4°C).

| β-lactams | Temperature | Mean | SD | N |
|---|---|---|---|---|
| 50 μg/mL ampicillin | 37°C | 3.21E+03 | 1.89E+03 | 3 |
| | 22°C (RT) | 1.30E+04 | 2.04E+03 | 3 |
| | 4°C | 8.87E+03 | 4.37E+03 | 3 |
| 50 μg/mL cephalothin | 37°C | 7.80E+04 | 1.23E+05 | 3 |
| | 22°C (RT) | 1.31E+05 | 1.15E+05 | 3 |
| | 4°C | 2.55E+05 | 3.15E+05 | 3 |

**Supplementary table 5**

Analysis of Variance, test effect of β-lactams (50 μg/mL ampicillin and 50 μg/mL cephalothin) and temperature (37°C, 22°C, and 4°C) on the evolutionary rescue of *E. coli*.

| | df | Sum Sq | Mean Sq | F value | Pr(>F) |
|---|---|---|---|---|---|
| antibiotics | 1 | 9.6208e+10 | 9.6208e+10 | 4.5142 | 0.05508 |
| temperature | 2 | 2.5849e+10 | 1.2925e+10 | 0.6064 | 0.56119 |
| antibiotics x temperature | 2 | 2.3841e+10 | 1.1921e+10 | 0.5593 | 0.58581 |
| Residuals | 12 | 2.5575e+11 | 2.1313e+10 | | |
